# Optical phase-truncation-based double-image encryption using equal modulus decomposition and random masks

**DOI:** 10.1038/s41598-024-57790-9

**Published:** 2024-03-26

**Authors:** Guangyu Luan, Chenggen Quan

**Affiliations:** 1 College of Electrical and Information, Heilongjiang Bayi Agricultural University, Daqing, 163319 Heilongjiang China; 2 Department of Mechanical Engineering, National University of Singapore, 9 Engineering Drive 1, Singapore, 117576 Singapore

**Keywords:** Electrical and electronic engineering, Information technology

## Abstract

This work reports an optical double-image crosstalk free encryption scheme that employs equal modulus decomposition and random masks. For the encryption, two plaintexts by a random amplitude mask and a random phase mask have been encrypted into a single ciphertext mask and two private key masks. Owing to the two random masks introduced, the functional relation between the plaintext pair and the ciphertext indirectly cause the paucity of constraints employed for the specific attack. Unlike the traditional phase-truncation-based techniques, this scheme is immune to the information leakage and different types of attacks. Furthermore, the three different diffraction distances and the illuminating wavelength also function as four additional keys to significantly reinforce the security. Simulation results demonstrate the feasibility and validity of the proposal.

## Introduction

Recently, optical techniques^1–11^ in image encryption have been intensely studied, owing to their inherent superiority with respect to multiple parameters and parallel processing. The pioneering work of optical image encryption is on double-random phase encoding (DRPE)^12^, which is done on the Fourier transform domain. Several works that followed have reported the expansion of initial DRPE work into different transform domains^13–17^, comprising the domains such as fractional Fourier transform (FrFT), Fresnel transform (FrT), and fractional random transform. During the period, other optical encryption works^18–30^ that employ compressive sensing, optical interference, iterative phase retrieval, digital holography, photon counting, and polarized light, have also emerged to improve the image security in succession.

However, owing to intrinsic linearity, DRPE-based structures cannot withstand several types of attacks^31–34^. Various nonlinear encryption methods have been proposed to overcome these weaknesses, and the best illustrative work is on phase-truncated Fourier transform by Qin and Peng^35^. Subsequently, there are several works based on phase truncation (PT) in FrT and FrFT domains^36–39^. Wang et al.^40^ showed that there is an information leakage in the work^35^ if one of two private keys are utilized, and hence, proposed a solution. They also pointed out the works^36–39^ being susceptible to information-leakage issue. Chen et al.^41^ developed a multi-image encryption scheme through feature fusion, compressed sensing, and PT. Yi and Tan^42^ presented a binary-tree multiple image encryption scheme. Su et al.^43^ proposed an optical encryption strategy for multiple color images through a complete trinary tree structure. Besides, the specific attack (SA)^44^, which employs an amplitude-phase-retrieval method, indicates that it can break the traditional PT-based technique^35^. Unfortunately, these techniques^41–43^ cannot be also an alternative to SA^44^. Additionally, the work^45^ has utilized the coherent superposition and equal modulus decomposition (EMD) to fully address the silhouette problem. However, there is the same modulus (i.e. same amplitude information) of two masks in EMD. The complex-valued masks cannot directly display in optical system. Chen et al.^46^ presented an optical image cryptosystem via two-beam coherent superposition and unequal amplitude decomposition for security improvement. However, unequal amplitude decomposition requires an additional random phase distribution than EMD. Thus, constantly improving the security of encryption scheme based on PT, despite the progress in the field, is still inevitable.

This study presents a double-image crosstalk free encryption scheme. The scheme, which works by utilizing the equal modulus decomposition and random masks, generates a single ciphertext mask and two private key masks. The random masks have been introduced to bring the indirect mathematical relation between the plaintext pair and the ciphertext, where an illegal user has insufficient constraints utilized for SA. Compared with certain PT-based works^35–39^, the proposal eliminates information leakage. When compared with other existing PT-based work^41–43^, the proposal is still effective against SA. The additional keys, which comprise the three different diffraction distances and the illuminating wavelength, can strengthen the security. Simulation results and performance analysis demonstrate the reliability and validity of the proposal.

## Principle of the method

**Figure 1:**
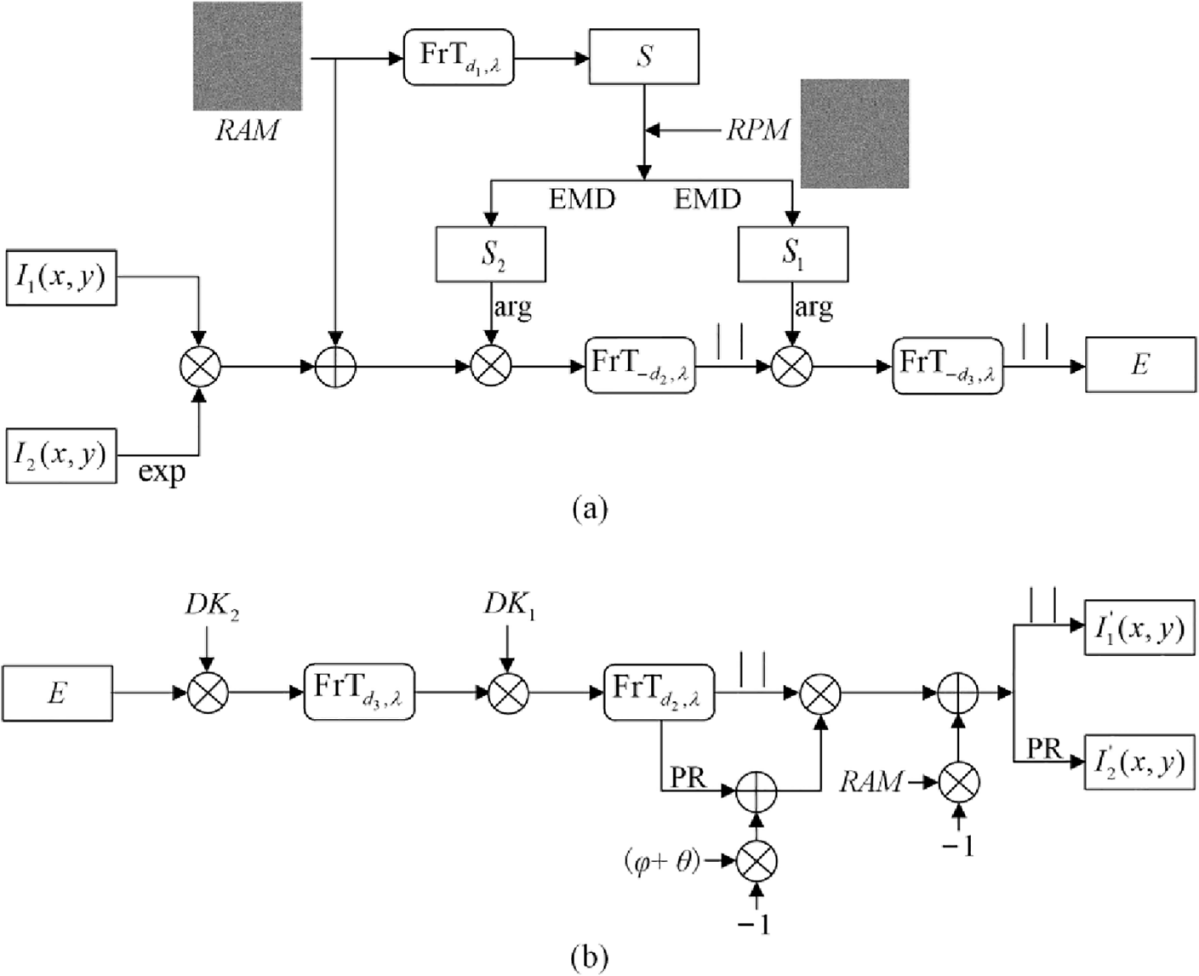
Schematic diagram for the proposed (**a**) encryption and (**b**) decryption.

**Figure 2:**
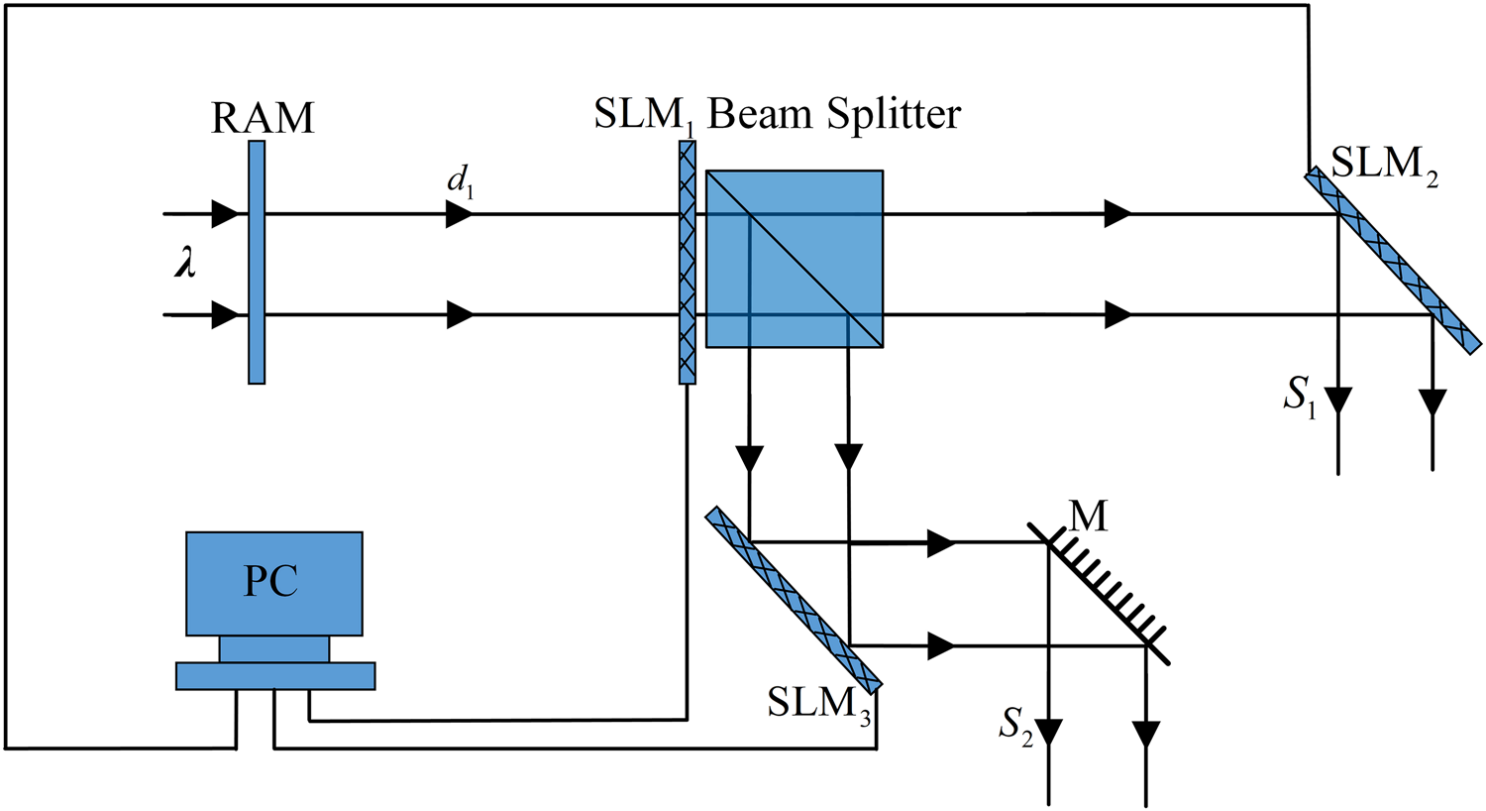
Optical relationship between RAM and *S*_1_, *S*_2_. *M* reflective mirrors.

The diagram of the proposed double-image cryptosystem is depicted as Fig. 1. The random amplitude mask (RAM) and a random phase mask (RPM) have been described with respect to the encryption (Fig. 1a) and decryption (Fig. 1b). The encryption keys have been generated from the random masks RAM and RPM by applying EMD in the Fresnel domain. Figure 2 shows the optical relationship between RAM, $S_{1}$, and $S_{2}$. In this setup, a collimated plane wave with the wavelength *λ,* perpendicularly illuminates RAM and the image which is subsequently Fresnel transformed with the diffraction distance *d*_1_. The first spatial light modulator (SLM_1_) were employed for amplitude modulation. SLM_2_ and SLM_3_ were utilized for phase modulation.

In the proposed double-image encryption process, $R_{1} (x,y)$ and $R_{2} \left( {u,v} \right)$ are the random functions, whose values fall in the interval [0, 1]. The following encryption steps have been depicted.

First, the RAM denoted as $R_{1} \left( {x,y} \right)$ was Fresnel transformed given by Eq. (1).

1 $$ S{ = }FrT_{{\left( {d_{1} ,\lambda } \right)}} \left[ {RAM} \right] = FrT_{{\left( {d_{1} ,\lambda } \right)}} \left[ {R_{1} \left( {x,y} \right)} \right] $$

where $FrT_{{(d_{1} ,\lambda )}} \left[ \cdot \right]$ is the FrT operator, and *d*_1_ and *λ* are the diffraction distance and the wavelength, respectively. The amplitude part and the phase part of *S* are represented as $A{ = }\left| S \right|$ and $\varphi = \arg \left[ S \right]$, respectively, and “$\left| \cdot \right|$” and “$\arg \left[ \cdot \right]$” are the modulus and argument operators, respectively.

**Figure 3:**
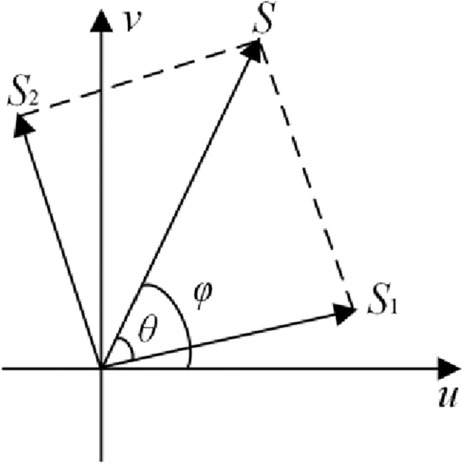
Principle of EMD.

Subsequently, the complex-valued function *S* was separated into two masks, viz, S_1_ and *S*_2_, with equal moduli, as illustrated in Fig. 3. Owing to the random phase distribution RPM (denoted as, $\theta (u,v)$) introduced, and the geometrical relationship, $S_{1}$ and $S_{2}$ can be deduced as

2 $$ \theta { = }RPM = 2\pi R_{2} \left( {u,v} \right) $$

3 $$ S_{1} { = }\frac{{{A \mathord{\left/ {\vphantom {A 2}} \right. \kern-0pt} 2}}}{\cos \left( \theta \right)}\exp [i\left( {\varphi - \theta } \right)] $$

4 $$ S_{2} { = }\frac{{{A \mathord{\left/ {\vphantom {A 2}} \right. \kern-0pt} 2}}}{\cos \left( \theta \right)}\exp [i\left( {\varphi + \theta } \right)] $$

The phase parts of $S_{1}$ and $S_{2}$ serve as the encryption keys.

Next, a new complex-value function $f(x,y)$ was constructed by the two original images, viz., ($I_{1} (x,y)$ and $I_{2} (x,y)$), and RAM, as follows

5 $$ f\left( {x,y} \right){ = }k_{{1}} I_{1} (x,y)\exp [ik_{{2}} I_{2} (x,y)] + k_{{3}} RAM(x,y) $$

where $k_{i} (i = 1,2,3)$ are the constants.

Then, by employing the encryption keys obtained in the above-mentioned step, the function $f(x,y)$ was encrypted based on PT in the Fresnel domain. Thus, the ciphertext $E$ can be expressed as

6 $$ E{ = }\left| {F{\text{rT}}_{{\left( { - d_{3} ,\lambda } \right)}} \left[ {\left| {F{\text{rT}}_{{\left( { - d_{2} ,\lambda } \right)}} \left[ {f\left( {x,y} \right)\exp \left[ {i(\varphi + \theta )} \right]} \right]} \right|\exp \left[ {i(\varphi - \theta )} \right]} \right]} \right| $$

where *d*_2_ and *d*_3_ are the diffraction distances. Meanwhile, two decryption keys, viz., $DK_{1}$ and $DK_{2}$, generated are given by

7 $$ DK_{1} {\text{ = conj}}\left\{ {\exp \left[ {i(\varphi - \theta )} \right]} \right\}PR\left[ {F{\text{rT}}_{{\left( { - d_{2} ,\lambda } \right)}} \left[ {f\left( {x,y} \right)\exp \left[ {i(\varphi + \theta )} \right]} \right]} \right] $$

8 $$ DK_{2} { = }PR\left[ {F{\text{rT}}_{{\left( { - d_{3} ,\lambda } \right)}} \left[ {\left| {F{\text{rT}}_{{\left( { - d_{2} ,\lambda } \right)}} \left[ {f\left( {x,y} \right)\exp \left[ {i(\varphi + \theta )} \right]} \right]} \right|\exp \left[ {i(\varphi - \theta )} \right]} \right]} \right] $$

where “${\text{conj}}\left\{ \cdot \right\}$” is the complex conjugate operator, $PR\left[ \cdot \right]$ the phase reservation operator.

For the decryption, the retrieved function $f{\prime} (x,y)$ by the authorized users can be derived as,

9 $$ f{\prime} \left( {x,y} \right){ = }\left| {F{\text{rT}}_{{\left( {d_{2} ,\lambda } \right)}} \left[ {F{\text{rT}}_{{\left( {d_{3} ,\lambda } \right)}} \left[ {DK_{2} E} \right]DK_{1} } \right]} \right|\exp \left[ {i\left[ {PR\left[ {F{\text{rT}}_{{\left( {d_{2} ,\lambda } \right)}} \left[ {F{\text{rT}}_{{\left( {d_{3} ,\lambda } \right)}} \left[ {DK_{2} E} \right]DK_{1} } \right]} \right] - \left( {\varphi + \theta } \right)} \right]} \right] $$

After obtaining the $f{\prime} (x,y)$, two retrieved images, $I_{1}{\prime} \left( {x,y} \right)$ and $I_{2}{\prime} \left( {x,y} \right)$, are mathematically represented as,

10 $$ I_{1}{\prime} \left( {x,y} \right){ = }\frac{{1}}{{k_{{1}} }}\left| {f{\prime} \left( {x,y} \right) - k_{{3}} RAM(x,y)} \right| $$

11 $$ I_{2}{\prime} \left( {x,y} \right){ = }\frac{{1}}{{k_{{2}} }}PR\left[ {f{\prime} \left( {x,y} \right) - k_{{3}} RAM(x,y)} \right] $$

**Figure 4:**
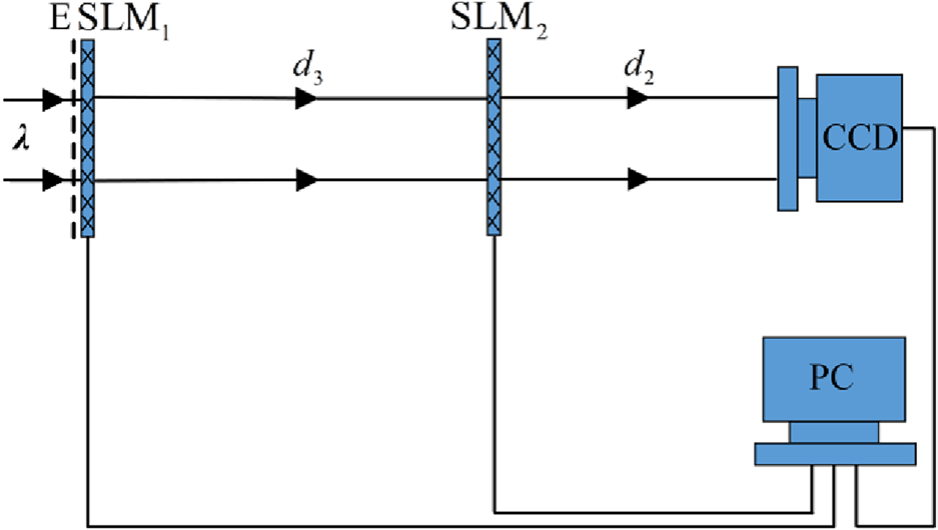
Optical schematic system for decryption.

Figure 4 illustrates the optical schematic apparatus for decryption. A light beam carrying the information of ciphertext *E* was modulated by SLM_1_. SLM_1_ and SLM_2_ are being employed for phase modulation. CCD captures the intensity part of $f{\prime} (x,y)$. The phase part of $f{\prime} (x,y)$ is digitally acquired. Then two decrypted images, viz.,$I_{1}{\prime} \left( {x,y} \right)$ and $I_{2}{\prime} \left( {x,y} \right)$, are digitally acquired with the function $f{\prime} \left( {x,y} \right)$.

## Numerical results and performance analysis

To demonstrate the validity and the advantages of the proposal, numerical simulations have been implemented. In those simulations, the illumination wavelength *λ* is 633 nm, and the three axial distances, viz., *d*_1_, *d*_2_, and *d*_3_, are 60, 50, and 70 mm, respectively, and the parameters *k*_1_, *k*_2_, and *k*_3_ are set as 0.6, 0.5, and 1.5, respectively. Furthermore, the correlation coefficient (CC) was utilized to objectively assess the similarity between the plaintext $I_{k} (x,y)$ (*k* = 1, 2) and its corresponding decrypted image $I_{k}{\prime} (x,y)$ as

12 $$ CC = \frac{{E\left\{ {\left[ {I_{k} (x,y) - E\left[ {I_{k} (x,y)} \right]} \right]} \right\}\left\{ {\left[ {I_{k}{\prime} (x,y) - E\left[ {I_{k}{\prime} (x,y)} \right]} \right]} \right\}}}{{E\sqrt {\left\{ {\left[ {I_{k} (x,y) - E\left[ {I_{k} (x,y)} \right]} \right]^{2} } \right\}} \sqrt {\left\{ {\left[ {I_{k}{\prime} (x,y) - E\left[ {I_{k}{\prime} (x,y)} \right]} \right]^{2} } \right\}} }} $$

For convenience of the analysis, the CC values were directly labelled in the recovered images.

**Figure 5:**
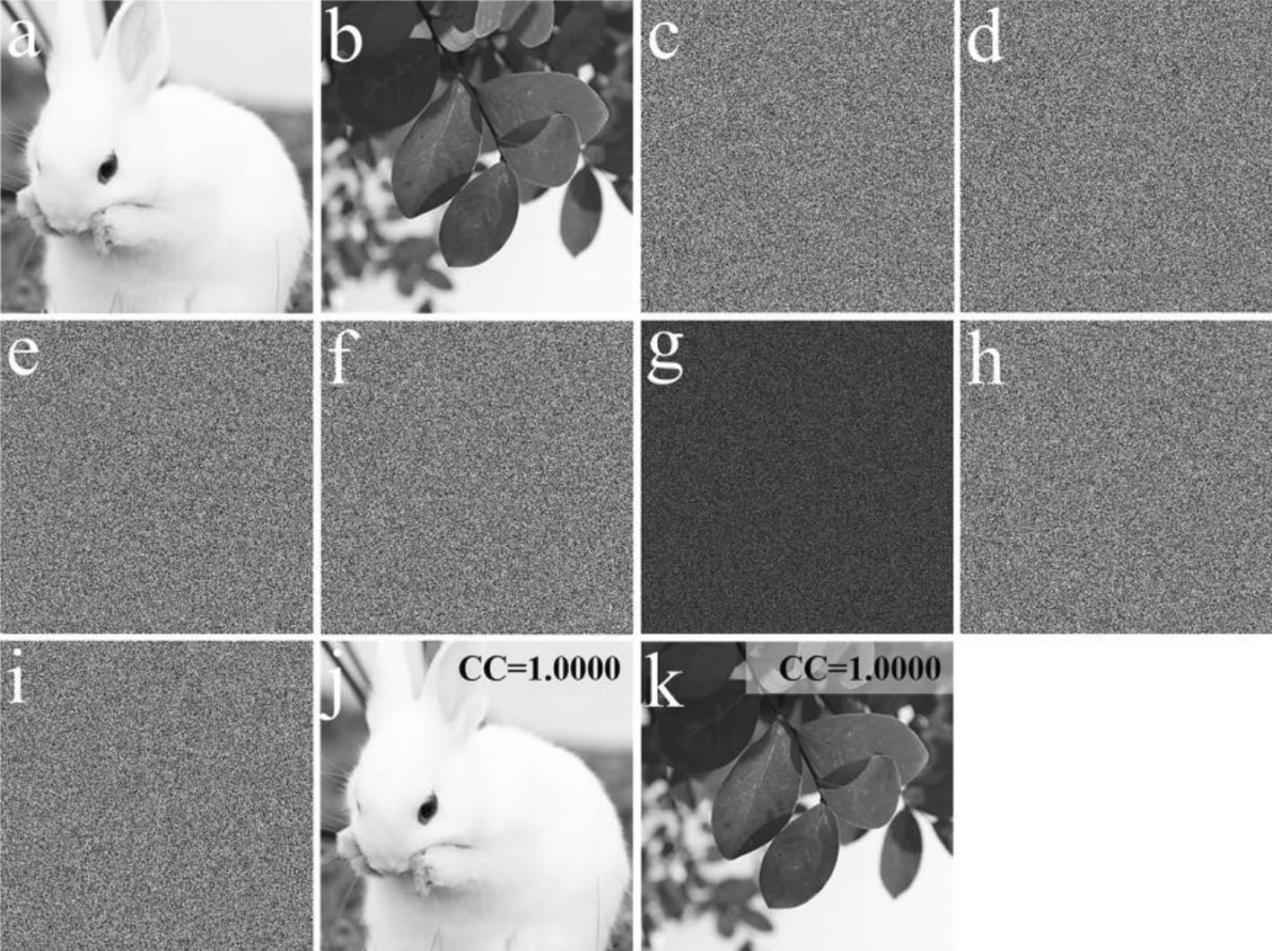
(**a**, **b**) The two plaintexts, (**c**) RAM, (**d**) RPM, (**e**, **f**) the two encryption keys, (**g**) the ciphertext *E*, (**h**) DK_1_, (**i**) DK_2_, (**j**, **k**) the two decrypted images.

Figure 5a, b show two original images having 512 × 512 pixels, which are employed as the two plaintexts. The two random masks, RAM and RPM, are shown in Fig. 5c, d. By employing RAM, RPM, and EMD, the encryption keys (Fig. 5e, f) are acquired. The ciphertext and decrypted keys, after conducting the proposed encryption process, are displayed in Fig. 5g–i, respectively. Finally, the decrypted images acquired by using all the correct keys are shown in Fig. 5j, k. These results signify that each decrypted image and its corresponding plaintext are completely equal, or the influence of crosstalk noise is non-existent. Thus, the proposal is feasible and effective, and can retrieve the high-quality images without the crosstalk noise.

**Figure 6:**
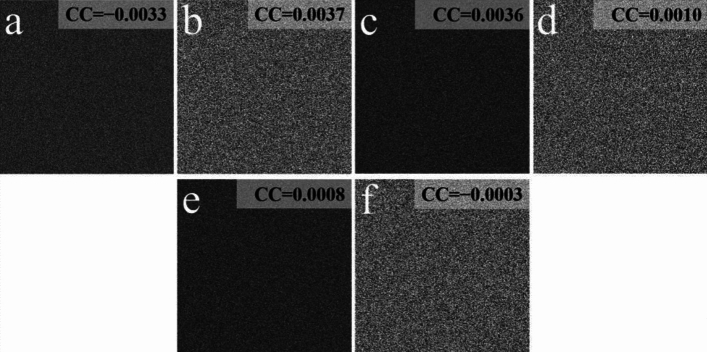
Decrypted images with (**a**, **b**) *E*, (**c**, **d**) *DK*_1_, and (**e**, **f**) *DK*_2_.

**Figure 7:**
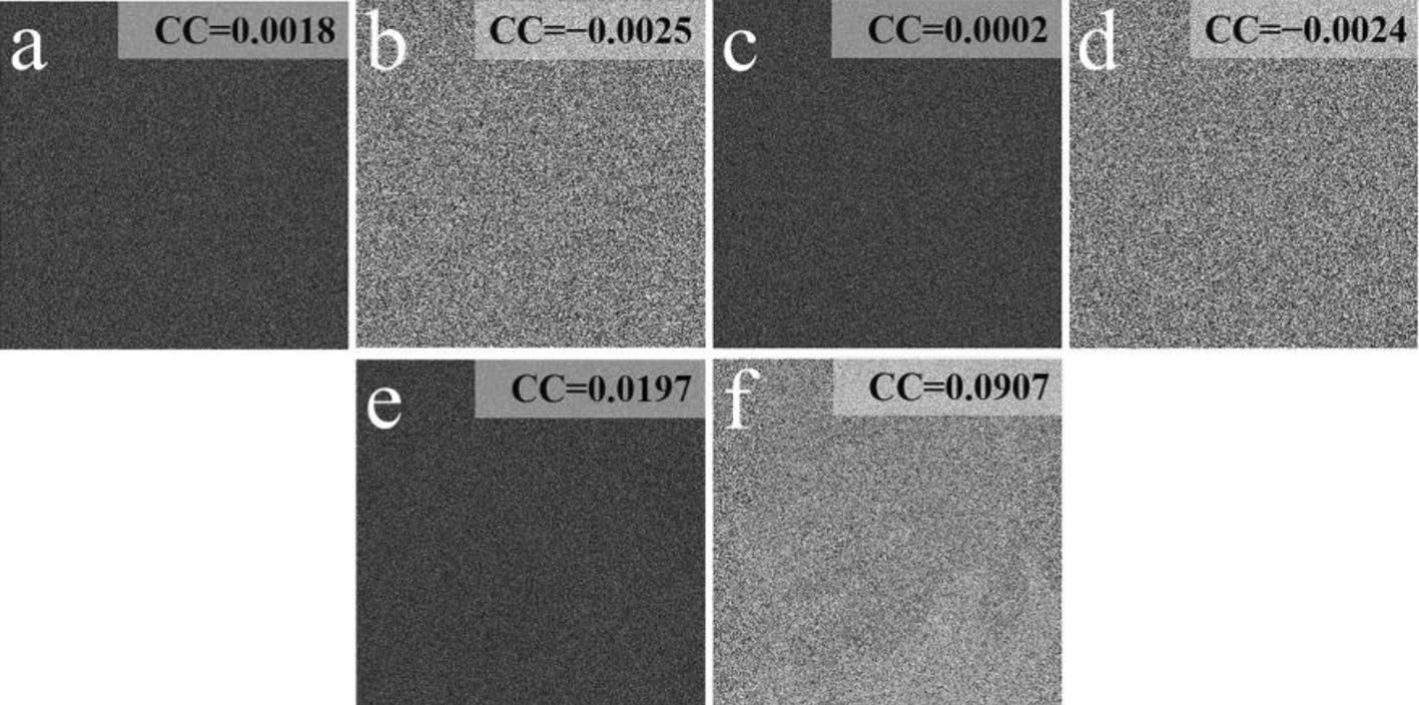
Decrypted images with (**a**, **b**) *E* and *DK*_1_, (**c**, **d**) *E* and *DK*_2_, and (**e**, **f**) *DK*_1_ and *DK*_2_.

To evaluate the information-leakage-free of the proposal, Fig. 6a–f show the decrypted images with releasing of *E*, *DK*_1_, and *DK*_2_. Figure 7a–f illustrate the decrypted images when two of these masks are utilized. According to Figs. 6 and 7, each of all the decrypted images has the noise-like distribution, where no useful information of the two plaintexts appears. We illustrated that the information-leakage issue has been thoroughly settled in the proposal.

**Figure 8:**
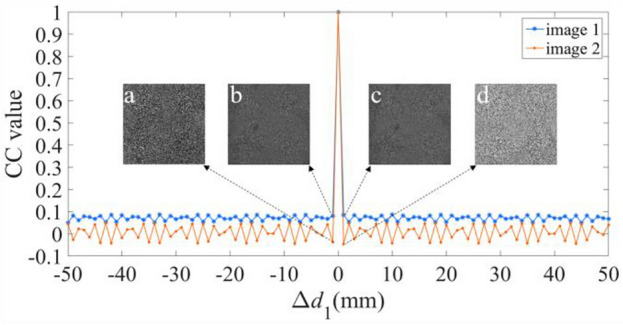
Relation curves of the CC value versus Δ*d*_1_, where the decrypted images using Δ*d*_1_ of (**a**, **b**) − 1, and (**c**, **d**) 1.

**Figure 9:**
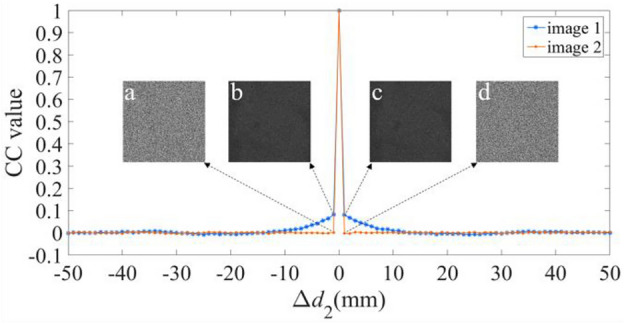
Relation curves of the CC value versus Δ*d*_2_, where the decrypted images using Δ*d*_2_ of (**a**, **b**) − 1, and (**c**, **d**) 1.

**Figure 10:**
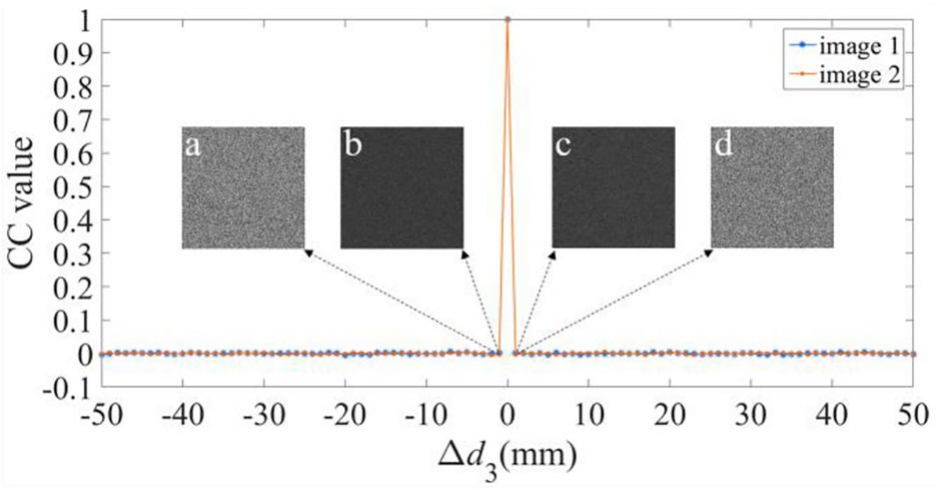
Relation curves of the CC value versus Δ*d*_3_, in which decrypted images using Δ*d*_3_ of (**a**, **b**) − 1, and (**c**, **d**) 1.

**Figure 11:**
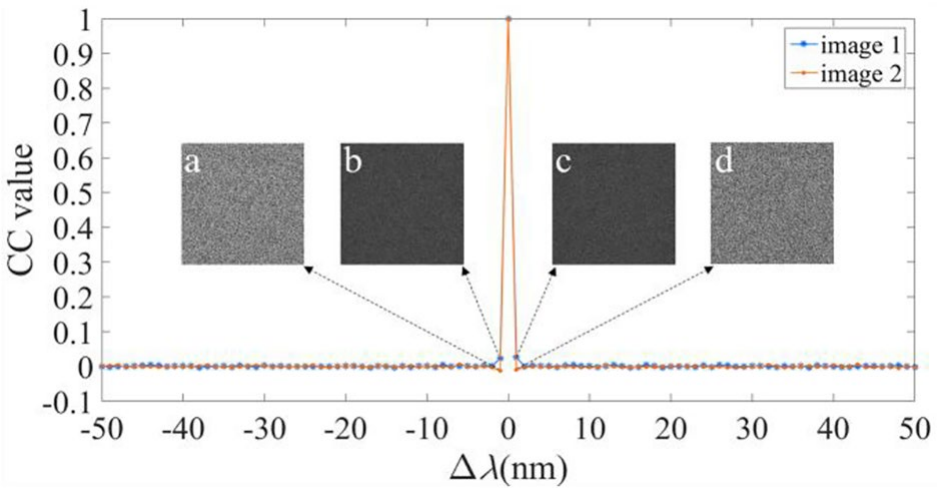
Relation curves of the CC value versus Δ*λ*, in which decrypted images using Δ*λ* of (**a**, **b**) − 1, and (**c**, **d**) 1.

We have further validated the sensitivity of the proposal for the additional keys, i.e., the three diffraction distances *d*_1_, *d*_2_, and *d*_3_, and the illuminating wavelength *λ*. Figures 8, 9, 10 and 11 illustrate the sensitive results of those keys, where the deviation ranges of those keys are [− 50 50]. These results invariably reveal that the CC values are one, only when the deviation is equal to zero. And for other values of the deviation, the CC values are below or equal to 0.0863. Moreover, when the deviations are − 1 and 1, all the decrypted images have no useful content of the two plaintexts. Therefore, the proposal has four sensitive additional keys, which can further reinforce the security of the proposal.

**Figure 12:**
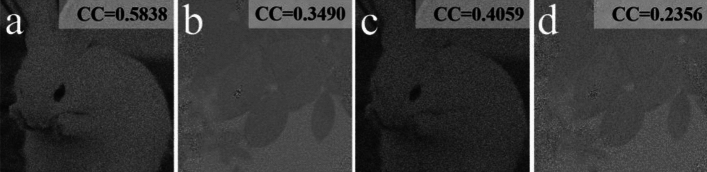
Decrypted images with Gaussian noise with (**a**, **b**) $\sigma = 0.2$, (**c**, **d**) $\sigma = 0.3$.

**Figure 13:**
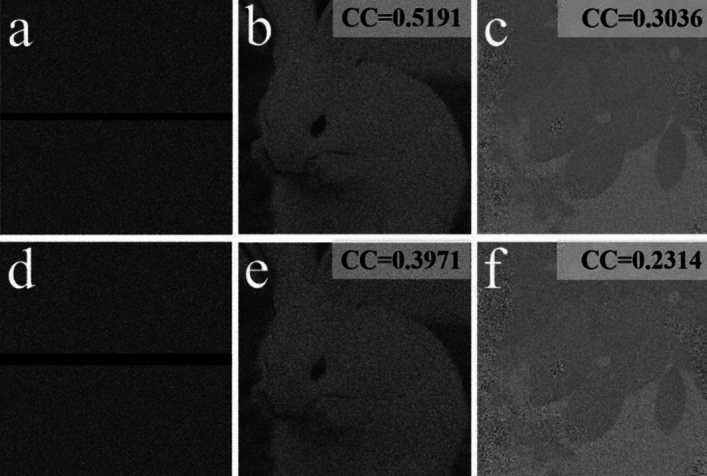
(**a**) Ciphertext with 3% occlusion, (**b**, **c**) decrypted images of (**a**, **d**) ciphertext with 5% occlusion, (**e**, **f**) decrypted images of (**d**).

To further demonstrate the robustness of the proposal against noise and occlusion attacks, Fig. 12 illustrates the decrypted results when the ciphertext is contaminated by the zero-mean white additive Gaussian noise with $\sigma = 0.2$ and $\sigma = 0.3$. Figure 13 represents the decrypted results acquired from the ciphertexts with 3% and 5% occlusion. According to Figs. 12 and 13, the quality of the decrypted images steadily worsens with the increase of the noise or occlusion strength. Although the content of those images becomes blurred for $\sigma = 0.3$ noise or 5% occlusion, the main-structure information can be still distinguished. Hence, it is verified for these results that the proposal has the resistance to noise and occlusion attacks.

**Figure 14:**
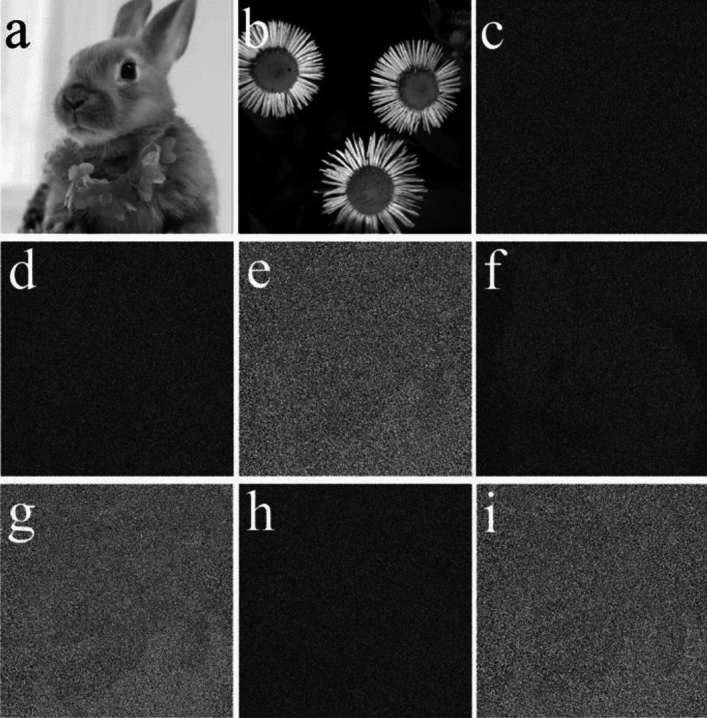
(**a**, **b**) the other plaintext pair, (**c**) ciphertext of (**a**, **b**) with RPM*'* and RAM*'*, retrieved images of (**c**) using (**d**, **e**) RAM, RPM, *DK*_1_, *DK*_2_, (**f**, **g**) RAM, RPM*'*, $DK_{1}^{{{\text{RPM}}^{\prime}}}$, $DK_{2}^{{{\text{RPM}}^{\prime}}}$, and (**h**, **i**) RAM*'*, RPM, $DK_{1}^{{{\text{RAM}}^{\prime}}}$, $DK_{2}^{{{\text{RAM}}^{\prime}}}$.

Furthermore, we have proved the validity of the proposal against known-plaintext attack (KPA). The decryption keys of our scheme varies with the different plaintext pair. In the KPA simulation, Fig. 5a, b, g serve as a known plaintext pair and their corresponding ciphertext. Using the proposal, RAM (Fig. 5c) and RPM (Fig. 5d) encrypt the plaintext pair in Fig. 5a, b, and the private keys in Fig. 5h, i are generated. Figure 14a, b demonstrate the other plaintext pair. Figure 14c illustrates the ciphertext of Fig. 14a, b, which were produced by the proposal, RAM′ and RPM′. RAM and RPM are different from RAM′ and RPM′, respectively. When all the correct parameters, viz., RAM (Fig. 5c), RPM (Fig. 5d), and the private keys (Fig. 5h, i) are used, the two retrieved results of Fig. 14c are shown in Fig. 14d, e. For better explaining the security of this scheme, we have retrieved Fig. 14c in the other two cases in which we suppose that the attacker has known one out of RAM′ and RPM′. Figure 14f, g demonstrate the results of Fig. 14c with RPM′, $DK_{1}^{{{\text{RPM}}^{\prime}}}$, $DK_{2}^{{\text{RPM}}\prime }$, and for the other abovementioned conditions. Figure 14h, i illustrate the results of Fig. 14c using RAM′, $DK_{1}^{{\text{RAM}}\prime }$, $DK_{2}^{{\text{RAM}}\prime }$, and the other conditions mentioned above. The information of Fig. 14a, b cannot be deciphered from Fig. 14d–i. Hence, the proposal is immune to KPA.

**Figure 15:**
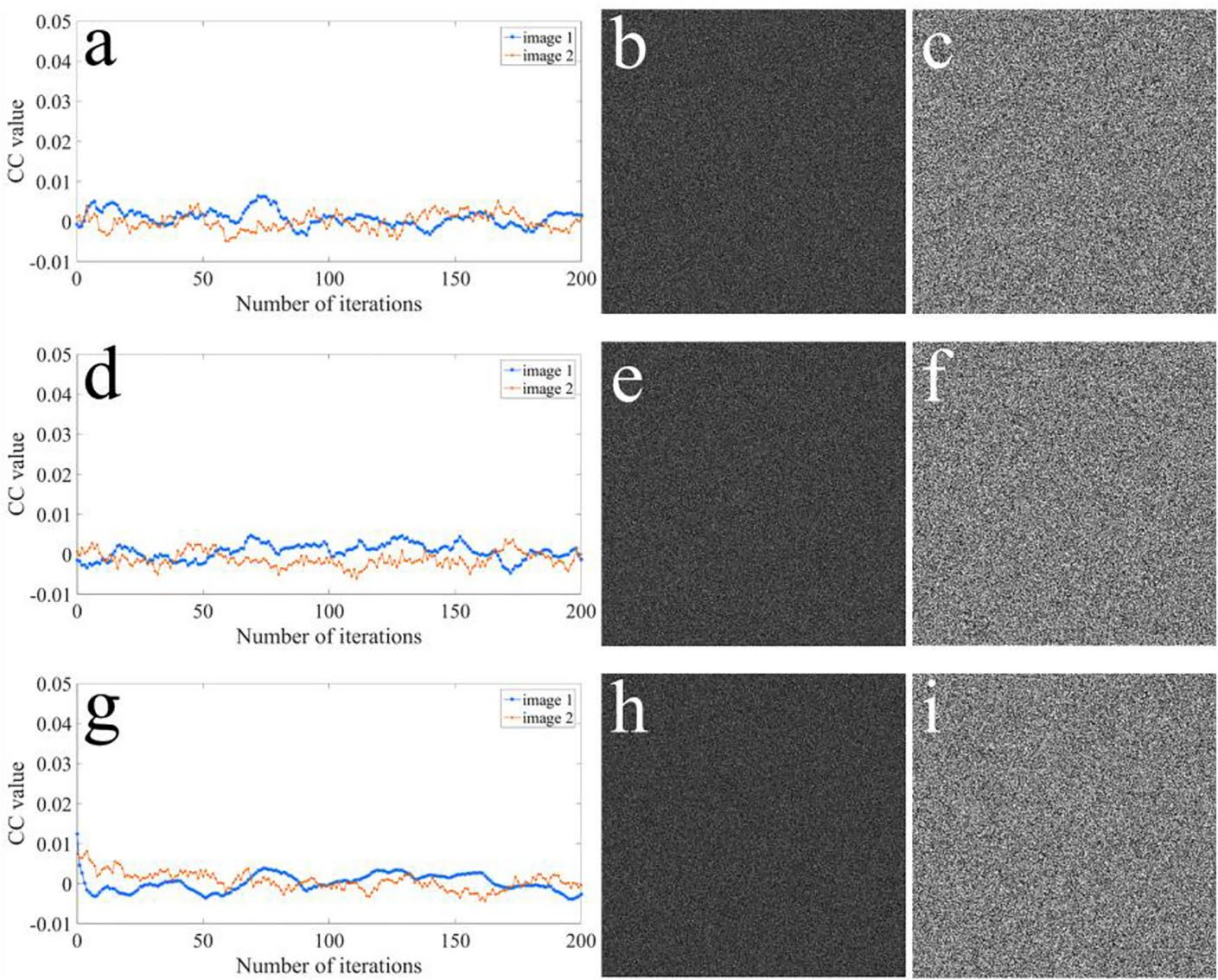
Results of SA using our proposal: the CC value versus number of iterations with (**a**) two unknown masks (RAM and RPM), (**d**) unknown RPM, and (**g**) unknown RAM, respectively; the recovered images after 200 iterations with (**b**, **c**) two unknown masks (RAM and RPM), (**e**, **f**) unknown RPM, and (**h**, **i**) unknown RAM, respectviely.

**Figure 16:**
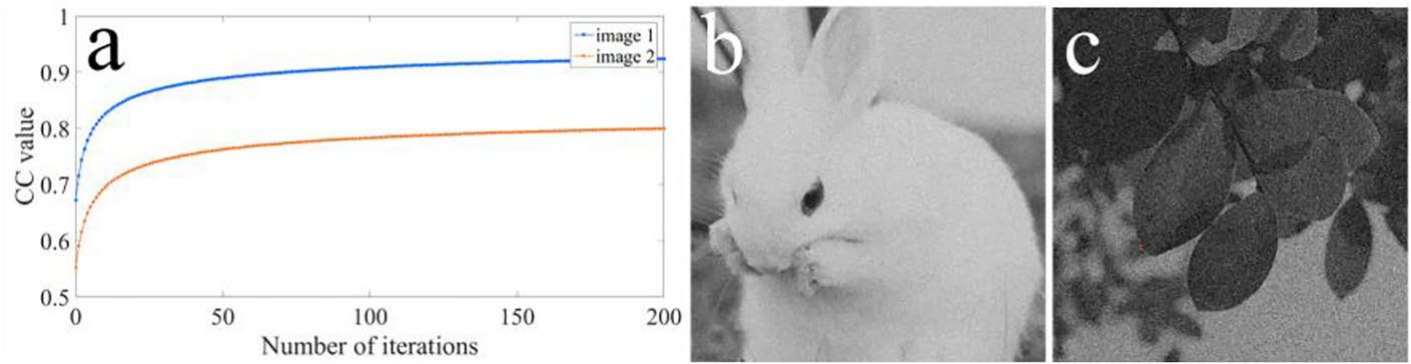
Results of SA using Qin and Peng’s scheme: (**a**) the CC value versus number of iterations, and (**b**, **c**) recovered images after 200 iterations.

Finally, we have also demonstrated that the proposal can resist SA^44^. SA is a single iteration process, which stem from the modified amplitude-phase retrieval algorithm. The results of SA with two unknown masks, viz., RAM and RPM, are illustrated in Fig. 15a–c. Moreover, the results of SA, when the knowledge of one of RAM and RPM is lacking, are illustrated in Fig. 15d–i. Figure 16 shows the results of SA using Qin and Peng’s scheme^35^. It is shown in Fig. 15a, d, g, that the six curves are unstable and non-convergent. According to Fig. 15b, c, e, f, h, i, no information can be deciphered of the two plaintexts (Fig. 15a, b). Therefore, we have shown that the proposal can effectively resist SA.

## Concluding remarks

An optical phase-truncation-based double-image encryption was developed using the equal modulus decomposition and random masks. The proposal utilizes RAM and RPM to generate a single ciphertext and two private keys. This scheme is novel, and acquires the decrypted images immune to the crosstalk noise. Particularly, the random masks cause insufficient constraints to be utilized for SA. Our proposal, when compared with the reported techniques via PT, has no problem of information leakage, and can efficiently resist different types of attacks. Furthermore, the four parameters serve as additional keys for enhancing the security. Numerical simulation results validate the advantages of the proposal.

## Data Availability

Data are available from the corresponding author upon reasonable request.

## References

[1] Alfalou A, Brosseau C (2009). Optical image compression and encryption methods. Adv. Opt. Photonics.

[2] Chen W, Javidi B, Chen XD (2014). Advances in optical security systems. Adv. Opt. Photonics.

[3] Javidi B (2016). Roadmap on optical security. J. Opt..

[4] Shikder A, Nishchal NK (2023). Image encryption using binary polarization states of light beam. Sci. Rep..

[5] Abuturab MR (2023). Multiple color image cryptosystem based on coupled-logistic-map-biometric keys, QR decomposition with column pivoting and optical Fresnel transform. Opt. Laser Technol..

[6] Wang XG, Chen W, Chen XD (2015). Optical encryption and authentication based on phase retrieval and sparsity constraints. IEEE Photonics J..

[7] Sui LS, Zhang LW, Wang Q, Tian AL, Anand A (2020). Multiple-image authentication based on the single-pixel correlated imaging and multiple-level wavelet transform. Opt. Lasers Eng..

[8] Wei HY, Wang XG (2021). Optical multiple-image authentication and encryption based on phase retrieval and interference with sparsity constraints. Opt. Laser Technol..

[9] Sui LS, Zhao XY, Huang CT, Tian AL, Anand A (2019). An optical multiple-image authentication based on transport of intensity equation. Opt. Lasers Eng..

[10] Qin Y, Jiang HL, Gong Q (2014). Interference-based multiple-image encryption by phase-only mask multiplexing with high quality retrieved images. Opt. Lasers Eng..

[11] Luan GY, Li AC, Zhang DM, Wang DX (2019). Asymmetric image encryption and authentication based on equal modulus decomposition in the Fresnel transform domain. IEEE Photonics J..

[12] Refregier P, Javidi B (1995). Optical image encryption based on input plane and Fourier plane random encoding. Opt. Lett..

[13] Li YB, Zhang F, Li YC, Tao R (2015). Asymmetric multiple-image encryption based on the cascaded fractional Fourier transform. Opt. Lasers Eng..

[14] Sui LS, Duan KK, Liang JL, Hei XH (2014). Asymmetric double-image encryption based on cascaded discrete fractional random transform and logistic maps. Opt. Express.

[15] Chen JX (2014). A novel double-image encryption scheme based on cross-image pixel scrambling in gyrator domains. Opt. Express.

[16] Wang XG, Chen W, Chen XD (2014). Fractional Fourier domain optical image hiding using phase retrieval algorithm based on iterative nonlinear double random phase encoding. Opt. Express.

[17] Xu HF, Xu WH, Wang SH, Wu SF (2017). Asymmetric optical cryptosystem based on modulus decomposition in Fresnel domain. Opt. Commun..

[18] Zhang Y, Wang B (2008). Optical image encryption based on interference. Opt. Lett..

[19] Perez-Cabre E, Cho MJ, Javidi B (2011). Information authentication using photon-counting double-random-phase encrypted images. Opt. Lett..

[20] Maluenda D, Carnicer A, Martinez-Herrero R, Juvells I, Javidi B (2015). Optical encryption using photon-counting polarimetric imaging. Opt. Express.

[21] Chen LF, Chang GJ, He BY, Mao HD, Zhao DM (2017). Optical image conversion and encryption by diffraction, phase retrieval algorithm and incoherent superposition. Opt. Lasers Eng..

[22] Li XW, Xiao D, Wang QH (2018). Error-free holographic frames encryption with CA pixel-permutation encoding algorithm. Opt. Lasers Eng..

[23] Carnicer A, Hassanfiroozi A, Latorre-Carmona P, Huang YP, Javidi B (2015). Security authentication using phase-encoded nanoparticle structures and polarized light. Opt. Lett..

[24] Rawat N, Hwang IC, Shi Y, Lee BG (2015). Optical image encryption via photon-counting imaging and compressive sensing based ptychography. J. Opt..

[25] Fatima A, Nishchal NK (2018). Optical image security using Stokes polarimetry of spatially variant polarized beam. Opt. Commun..

[26] Wang Y, Quan C, Tay CJ (2016). Asymmetric optical image encryption based on an improved amplitude-phase retrieval algorithm. Opt. Lasers Eng..

[27] Moon I, Yi F, Han M, Lee J (2016). Efficient asymmetric image authentication schemes based on photon counting-double random phase encoding and RSA algorithms. Appl. Opt..

[28] Chen Y, Liu Q, Wang J, Wang QH (2017). Single-channel optical encryption of color image using chessboard grating and diffraction imaging scheme. Opt. Eng..

[29] Su YG (2017). Cascaded Fresnel holographic image encryption scheme based on a constrained optimization algorithm and Henon map. Opt. Lasers Eng..

[30] Qin Y, Wang ZP, Wang HJ, Gong Q, Zhou NR (2018). Robust information encryption diffractive-imaging-based scheme with special phase retrieval algorithm for a customized data container. Opt. Lasers Eng..

[31] Gopinathan U, Monaghan DS, Naughton TJ, Sheridan JT (2006). A known-plaintext heuristic attack on the Fourier plane encryption algorithm. Opt. Express.

[32] Peng X, Zhang P, Wei HZ, Yu B (2006). Known-plaintext attack on optical encryption based on double random phase keys. Opt. Lett..

[33] Tashima H (2010). Known plaintext attack on double random phase encoding using fingerprint as key and a method for avoiding the attack. Opt. Express.

[34] Zhang YS, Xiao D, Wen WY, Liu H (2013). Vulnerability to chosen-plaintext attack of a general optical encryption model with the architecture of scrambling-then-double random phase encoding. Opt. Lett..

[35] Qin W, Peng X (2010). Asymmetric cryptosystem based on phase-truncated Fourier transforms. Opt. Lett..

[36] Chen W, Chen XD (2011). Optical color image encryption based on an asymmetric cryptosystem in the Fresnel domain. Opt. Commun..

[37] Rajput SK, Nishchal NK (2012). Asymmetric color cryptosystem using polarization selective diffractive optical element and structured phase mask. Appl. Opt..

[38] Rajput SK, Nishchal NK (2012). Image encryption based on interference that uses fractional Fourier domain asymmetric keys. Appl. Opt..

[39] Mehra I, Rajput SK, Nishchal NK (2013). Collision in Fresnel domain asymmetric cryptosystem using phase truncation and authentication verification. Opt. Eng..

[40] Wang XG, Zhao DM, Chen YX (2014). Double-image encryption without information disclosure using phase-truncation Fourier transforms and a random amplitude mask. Appl. Opt..

[41] Chen XD, Liu Q, Wang J, Wang QH (2018). Asymmetric encryption of multi-image based on compressed sensing and feature fusion with high quality image reconstruction. Opt. Laser Technol..

[42] Yi JW, Tan GZ (2016). Binary-tree encryption strategy for optical multiple-image encryption. Appl. Opt..

[43] Su YG (2017). Optical encryption scheme for multiple color images using complete trinary tree structure. Opt. Lasers Eng..

[44] Wang XG, Chen YX, Dai CQ, Zhao DM (2014). Discussion and a new attack of the optical asymmetric cryptosystem based on phase-truncated Fourier transform. Appl. Opt..

[45] Cai J, Shen X, Lei M, Lin C, Dou S (2015). Asymmetric optical cryptosystem based on coherent superposition and equal modulus decomposition. Opt. Lett..

[46] Chen LF, Gao X, Chen XD, He BY, Liu JY, Li D (2016). A new optical image cryptosystem based on two-beam coherent superposition and unequal modulus decomposition. Opt. Laser Technol..

